# *Tbx1* and *Jag1* act in concert to modulate the fate of neurosensory cells of the mouse otic vesicle

**DOI:** 10.1242/bio.027359

**Published:** 2017-08-24

**Authors:** Stephania Macchiarulo, Bernice E. Morrow

**Affiliations:** 1 Department of Genetics, Albert Einstein College of Medicine, 1301 Morris Park Avenue, Bronx, NY 10461, USA; 2 Departments of Obstetrics and Gynecology and Pediatrics, Albert Einstein College of Medicine, 1301 Morris Park Avenue, Bronx, NY 10461, USA

**Keywords:** Otic vesicle, Inner ear, Notch, Tbx1, Jag1, Neurosensory

## Abstract

The domain within the otic vesicle (OV) known as the neurosensory domain (NSD), contains cells that will give rise to the hair and support cells of the otic sensory organs, as well as the neurons that form the cochleovestibular ganglion (CVG). The molecular dynamics that occur at the NSD boundary relative to adjacent OV cells is not well defined. The *Tbx1* transcription factor gene expression pattern is complementary to the NSD, and inactivation results in expansion of the NSD and expression of the Notch ligand, Jag1 mapping, in part of the NSD. To shed light on the role of *Jag1* in NSD development, as well as to test whether *Tbx1* and *Jag1* might genetically interact to regulate this process, we inactivated *Jag1* within the *Tbx1* expression domain using a knock-in *Tbx1^Cre^* allele. We observed an enlarged neurogenic domain marked by a synergistic increase in expression of *NeuroD* and other proneural transcription factor genes in double *Tbx1* and *Jag1* conditional loss-of-function embryos. We noted that neuroblasts preferentially expanded across the medial-lateral axis and that an increase in cell proliferation could not account for this expansion, suggesting that there was a change in cell fate. We also found that inactivation of *Jag1* with *Tbx1^Cre^* resulted in failed development of the cristae and semicircular canals, as well as notably fewer hair cells in the ventral epithelium of the inner ear rudiment when inactivated on a *Tbx1* null background, compared to *Tbx1^Cre/−^* mutant embryos. We propose that loss of expression of *Tbx1* and *Jag1* within the *Tbx1* expression domain tips the balance of cell fates in the NSD, resulting in an overproduction of neuroblasts at the expense of non-neural cells within the OV.

## Abstract (precis)

**Summary:** Normal dosages of *Tbx1* and *Jag1* are required to maintain a proper balance of cell types within the neurosensory domain of the otic vesicle to form the inner ear.

## INTRODUCTION

The inner ear forms from the otic vesicle (OV), which is a closed, continuous epithelium consisting of cells that will differentiate to form specialized cell types (Bok et al., 2007). The T-box transcription factor gene, *Tbx1*, plays a major role in the OV for inner ear morphogenesis. *Tbx1* is expressed in the posterior-lateral OV, mostly complementary to the region that forms the cochleovestibular ganglion (CVG) (Ma et al., 1998; Raft et al., 2004). When *Tbx1* is inactivated in the mouse, the CVG is duplicated in size, marked by an expansion in expression of *Atonal*-related basic helix-loop-helix (bHLH) proneural transcription factors *Neurogenin1* (*Ngn1*; also known as *Neurog1*) and *NeuroD* (*Neurod1*), in the neurogenic domain (Raft et al., 2004). In addition, the cochlea and vestibular system do not develop (Vitelli et al., 2003; Raft et al., 2004). However, failure of otic epithelial cells to proliferate normally in *Tbx1* null mice may render it difficult to identify the range of its specific requirements (Xu et al., 2007). Overexpression (Funke et al., 2001) or constitutive expression of *Tbx1* (Freyer et al., 2013), on the other hand, has the opposite effect for neurogenesis, meaning that the neurogenic domain is reduced in area (Freyer et al., 2013). In addition to this defect, the utricle and the saccule, containing hair cells derived from prosensory patches during early development, do not form. This suggests that *Tbx1* may restrict neurogenesis and sensorigenesis. We are interested in understanding the mechanism for this, so we considered other genes that are required for these processes.

The Notch ligand, Jagged1 (Jag1), and group B Sox SRY-related HMG box transcription factor (Sox2) are broadly co-expressed in the ventral region of the OV. Within these expression domains, exists a subdomain known as the neurosensory domain (NSD), the cells of which have the potential to differentiate into neurons, hair cells or support cells (Adam et al., 1998; Cole et al., 2000; Daudet et al., 2007; Fekete and Wu, 2002; Kiernan et al., 2006, 2005; Morsli et al., 1998; Neves et al., 2011; Pan et al., 2010; Raft and Groves, 2014). Mouse genetic studies show that inactivation of *Jag1* results in failed development of prosensory patches early in development, and a reduction in the size of the neurogenic zone of the CVG (Kiernan et al., 2006; Pan et al., 2010), suggesting that it may be required for neurosensory competence or maintenance. Sox2 expression is maintained by *Jag1* and is also required for neurosensory specification (Kiernan et al., 2006; Neves et al., 2011; Pan et al., 2010). Tbx1 appears to function in a complementary manner to Jag1.

Little is known about the cellular and molecular dynamic requirements of cells in the NSD for proper inner ear development. Notch pathway genes have been implicated in tissue boundary formation during development in various contexts. Since loss of *Tbx1* results in an expansion of the CVG and inactivation of *Jag1* results in a somewhat smaller CVG (Kiernan et al., 2006; Pan et al., 2010), we wanted to test whether the two genes might act antagonistically in the NSD for inner ear development. In this study, we utilized *Tbx1* (Huynh et al., 2007) and *Jag1* (Kiernan et al., 2006) conditional loss-of-function mouse mutants to evaluate their functions in the NSD, and found a surprising novel function in neurogenesis.

## RESULTS

### Expression of Tbx1 and Jag1 around the NSD

**Fig. 1.**
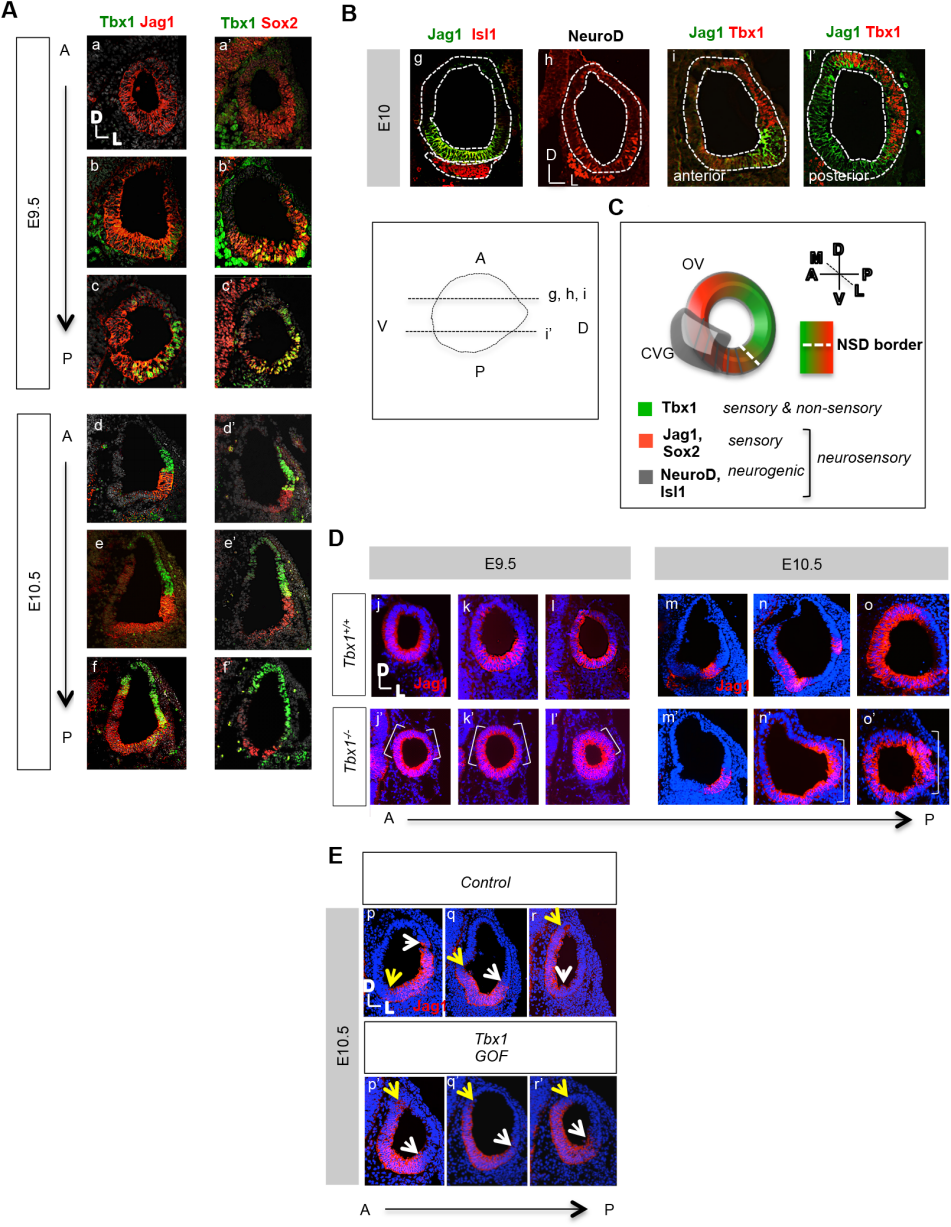
**NSD gene expression in wild-type and *Tbx1* null embryos.** (A) Immunofluorescence was performed using antibodies to Jag1, Tbx1 and Sox2 on tissue sections from mouse embryos at E9.5 (a-c′) and E10.5 (d-f′). Jag1 and Sox2 are expressed broadly in the otic epithelium at E9.5, overlapping with Tbx1 expression in the PVL OV, but are largely complementary. By E10.5, Jag1 and Sox2 expression is mainly restricted to the anterior ventral lateral and posterior medial OV, with less overlap with Tbx1 expression, which is restricted to the posterior dorsal lateral OV. (B) Immunofluorescence was performed using antibodies to Jag1, Tbx1, Isl1 and NeuroD, as indicated (g-i′). Jag1 is expressed in the NSD, where neuroblasts expressing both Isl1 (g) and NeuroD (h) are derived. Tbx1 is expressed complementary to Jag1 (i,i′), as well as NeuroD (h), at the same border. The diagram below depicts the planes of sections used. (C) Schematic summarizing the wild-type expression of the genes examined in B, with respect to domains. (D) Immunofluorescence for Jag1 (red) on transverse sections in embryos at E9.5 and E10.5 was performed. The sections progress from the anterior to posterior domains of the OV. At E9.5, Jag1 expression expands in both the medial and lateral domain (white brackets), while at E10.5, Jag1 expression expands in the lateral domain (white brackets). (E) Immunofluorescence for Jag1 in *Tbx1 GOF* and littermate control embryos showing a shift in the expression from lateral (white arrow) to medial (yellow arrow) in anterior sections (p′,q′).

Both *Jag1* and *Sox2* are markers of the NSD-containing cells required for neurogenesis and sensorigenesis (Raft and Groves, 2014). We performed immunofluorescence studies to determine where and when Tbx1, Jag1 and Sox2 are expressed with respect to each other and to the NSD (Fig. 1). At embryonic day (E) 9.5, Tbx1 was broadly expressed in the posterolateral wall of the OV (Fig. 1A, b-c′; Fig. S1). As expected, Jag1and Sox2 proteins were expressed in a similar pattern to one another. Expression of the two was complementary to Tbx1 in the anterior domain, while there was some overlap in expression with Tbx1 in the posterior lateral OV (Fig. 1A, a-c′). By E10.5, expression of all three genes became more restricted within their respective complementary domains, such that Jag1 and Sox2 had less overlap in expression with Tbx1 (Fig. 1A, d-f; Fig. S1). The complementary expression patterns between Tbx1 and Sox2/Jag1 and known loss-of-function phenotypes (Raft et al., 2004; Pan et al., 2010; Puligilla et al., 2010) suggest that there might be an antagonistic relationship between them. We decided to focus on evaluating the relationship between Tbx1 and Jag1 in more detail, since there appeared to be a sharper border between Jag1 and Tbx1, than Sox2 and Tbx1, expression (Fig. 1A), suggesting that the two may interact genetically to form an important boundary within the OV. Another reason to focus our study on *Jag1* is that *Jag1* has been shown to function upstream of *Sox2* during inner ear development (Pan et al., 2010; Neves et al., 2011). Finally, *Notch1* has been shown to function downstream of *Tbx1* during neurogenesis in the OV (Xu et al., 2007), providing additional rationale to further study its ligand, *Jag1*, which had never been linked to *Tbx1* up until this point.

At this point, in order to evaluate Tbx1 and Jag1 expression with respect to regions of neurogenesis, the expression of Jag1, Tbx1, Isl1, and NeuroD were compared (Fig. 1B). Jag1 was expressed in the region where neuroblasts expressing both Isl1 (Fig. 1B, g) and NeuroD (Fig. 1B, h) are derived, while Tbx1 was expressed in a largely complementary manner to these genes (Fig. 1B, i,i′). This indicates that Tbx1 is excluded from the neurogenic domain within the NSD, marked by overlapping expression of Jag1 and NeuroD. A schematic of expression patterns is shown in Fig. 1C, showing the expression patterns of these genes, with respect to one another and the NSD border.

We also wanted to determine what effect loss or gain of *Tbx1* would have on Jag1 expression and, indirectly, the NSD. At E9.5, *Jag1* expression expanded anteriorly and posteriorly on the medial and lateral walls of the OV when *Tbx1* was globally inactivated (Fig. 1D, j′,k′,l′). By E10.5, *Jag1* expression in wild-type embryos became restricted mostly to the posteromedial OV, with small regions of expression in the anterolateral wall of the OV (Fig. 1D, m,n,o). When *Tbx1* was inactivated, *Jag1* expression again expanded across the medial-lateral axis of the posterior OV (Fig. 1D, m′,n′,o′). The expansion in *Jag1* expression into the posterior OV correlated with the region of ectopic neurogenesis in *Tbx1*^−/−^ OVs that has been previously described (Fig. 1B) (Raft et al., 2004; Xu et al., 2007). We also examined the expression of *Jag1* in *Tbx1 GOF* (*Pax2-Cre/+; Tbx1-GFP/+*) (Freyer et al., 2013) embryos (Fig. 1E), and found that the expression domain shifted from the anteroventral lateral to anteroventral medial OV (Fig. 1E, p,p′). In control embryos, Jag1 is expressed in the medial OV in more posterior sections (Fig. 1E, q,r), whereas in in anterior sections, Jag1 expression is exclusively lateral (Fig. 1E, p). In contrast, in *Tbx1 GOF* embryos, Jag1 expression is almost exclusively medial throughout the OV, with significantly more coverage of the medial wall of the OV compared to controls, particularly in anterior sections (Fig. 1E, p′,q′,r′). These findings indicate a change in the position of the medial-lateral border and shift of the NSD. Of note, cells within the anteroventral lateral region of the OV contribute to the CVG and utricle (Fekete and Wu, 2002), the cells of which are derived from a shared lineage within the NSD (Raft et al., 2007) We further asked whether there was mutual antagonism between *Tbx1* and *Jag1*, by examining Tbx1 expression in *Jag1* null mice in the OV. There was some decrease in the area of Tbx1 expression, although this loss is likely secondary to the reduction in size of the OV (Fig. S2). The position of the expression domain with respect to the OV appeared unaltered. These findings suggest that there is a unidirectional pathway by which *Tbx1* restricts the Jag1 expression and the size and location of the NSD.

**Fig. 2.**
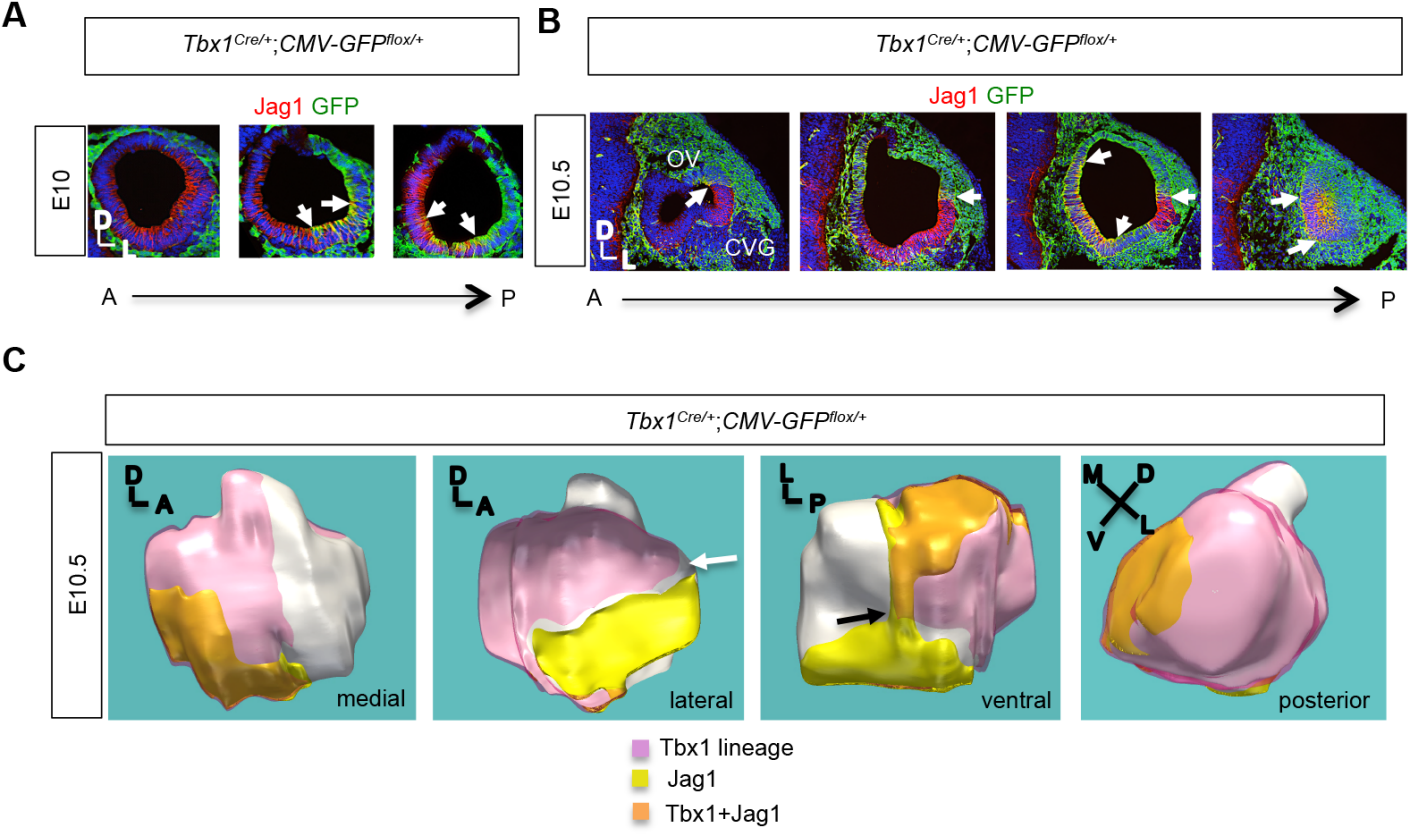
***Tbx1* lineage is largely complementary to the NSD.** (A,B) Immunofluorescence for Jag1 (red) and GFP (green) on transverse sections of a *Tbx1^Cre/+^;CMV-GFP ^flox/+^* embryo at E10 and E10.5, showing largely complementary expression between Jag1 and the Tbx1 cell lineage in more anterior sections of the OV, but some colocalization (white arrows) in more posterior ventral regions. (C) 3D reconstruction of serial sections of the E10.5, *Tbx1^Cre/+^;CMV-GFP ^flox/+^* embryo shown in Fig. 3C, showing the position of expression of the *Tbx1* lineage and Jag1 as well as co-expression of both genes. The *Tbx1* cell lineage is shown in pink, Jag1 protein expression is shown in yellow, and the overlap between the two is shown in orange. A dorsal-ventral (white arrow) and medial-lateral (black arrow) border between the *Tbx1* lineage and Jag1 expression is shown.

**Fig. 3.**
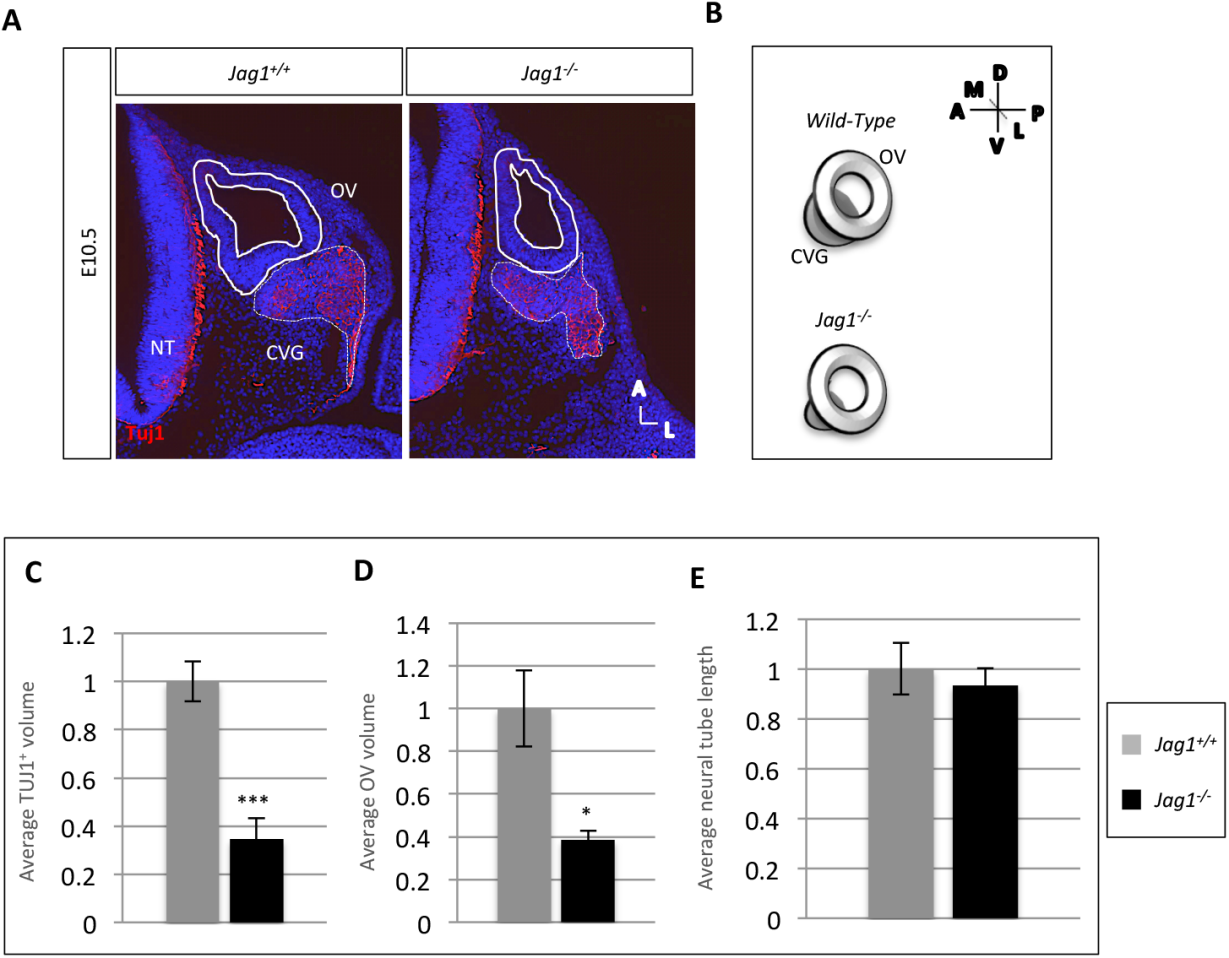
**The CVG is significantly smaller in size in *Jag1^−/−^* embryos compared to wild-type controls at E10.5.** (A) Immunofluorescence using a Tuj1 antibody was performed to mark the CVG (traced with a white dashed line). (B) Schematic depicting the phenotype in A. (C) The average volume of the CVG marked by Tuj1 expression was measured and averaged per ear per genotype. Average OV volume was also calculated. Both CVG and OV volume in *Jag1^−/−^* embryos were significantly lower than in *Jag1^+/+^* embryos. Average neural tube length was also calculated to serve as a control for overall embryo size; there was no significant difference between genotypes. (*Jag1^+/+^* embryos, *n*=3; *Jag1^−/−^* embryos, *n*=4). ***P<0.001; *P<0.05. Data are mean±s.e.m. Values were normalized to those of wild-type levels.

Next, we performed cell lineage tracing of *Tbx1* using a *Tbx1^Cre^* mouse line (Huynh et al., 2007) crossed with a GFP reporter line (Batista-Brito et al., 2009) in order to map the overlap between the *Tbx1* lineage and Jag1 expression to know where *Jag1* might be getting inactivated. We found that Jag1 partially colocalized with the *Tbx1* cell lineage, marked by GFP in mostly the posterior ventral lateral (PVL) OV at E10 (Fig. 2A), but broadened to include the posterior medial domain at E10.5 (Fig. 2B). There was less overlap in the anteroventral region, adjacent to the CVG (Fig. 3A-C). On a separate note, there is another distinct *Tbx1* lineage within the mesoderm surrounding the OV as well (Fig. 2B). The OV from the E10.5 embryo shown in Fig. 2B was used for serial section 3D reconstruction (Fig. 2C). The purpose was to better visualize the areas of overlap between Jag1 and the *Tbx1* cell lineage. This clarified that Jag1 expression occurred mostly at the border of the *Tbx1* lineage, with overlap occurring primarily in the PVL and posterior ventral medial (PVM) domains, which contribute to the lateral SCC and, in part, the saccule and cochlea, respectively (Fig. 2C) (Fekete and Wu, 2002). A very clear border between the *Tbx1* lineage and *Jag1* expression can been seen at the dorsal-ventral (white arrow) and medial-lateral (black arrow) axis (Fig. 2C). We also performed *Tbx1* lineage tracing when *Tbx1* was homozygously inactivated, in conjunction with antibody staining for NeuroD. Interestingly, this revealed that while the ectopic CVG that forms in *Tbx1* null embryos is composed largely of cells derived from the *Tbx1* cell lineage, a number of neuroblasts positive for NeuroD are GFP-negative (GFP^−^), suggesting they are not derived from the *Tbx1* lineage and could indicate nonautonomous roles (Fig. S3). Previous findings that Jag1-Notch1 signaling plays a role in CVG formation (Pan et al., 2010) and loss of *Tbx1* affects aNotch expression (Xu et al., 2007), provide a reasonable basis for speculating that *Tbx1* may regulate CVG formation nonautonomously by affecting this cell signaling pathway. Another point worth noting is that it has been previously shown (and confirmed in this report) that the CVG and distal ganglia fuse in *Tbx1* null embryos (Xu et al., 2007). Thus, one might speculate that GFP^−^ cells from this experiment are derived from epibranchial ganglia; however, these authors also show contribution of the *Tbx1* cell lineage to epibranchial ganglia, particularly the IXth ganglion, when one allele of *Tbx1* is inactivated (Xu et al., 2007). Thus, some GFP^+^ cells in this experiment may also derive from epibranchial ganglia.

To further delineate Tbx1 and Jag1 domains with respect to the NSD border, we also performed lineage tracing using a *Pax3^C^^re^* (*Paired Box Gene 3*) mouse line crossed with GFP reporter mice to mark the neural crest cell lineage. This lineage is present in the neurosensory regions of the inner ear, except for the cristae (Freyer et al., 2011). Immunofluorescence on alternating serial sections at E9.5 demonstrated that Jag1 was expressed throughout much of the *Pax3* lineage (Fig. S4, top; white brackets), while Tbx1 was complementary to this (Fig. S4, bottom; white arrowheads). When taken together, these data show that *Tbx1* expression borders and restricts *Jag1* expression, primarily in regions within the NSD that give rise to the utricle and CVG.

### Inactivation of *Jag1* results in a significant reduction in the CVG size

We evaluated E10.5 *Jag1^−/−^* versus *Jag1^+/+^* embryos using an anti-Tuj1 antibody, which marks differentiated neurons. We measured the area around the Tuj1 staining marking the CVG and found that there was a significant decrease in the average area of the CVG in *Jag1* null embryos compared to wild-type controls, to ∼50% of wild-type levels (*P*<0.001) (Fig. 3). This is a more drastic reduction than previously reported in *Jag1* conditional loss-of-function embryos (Pan et al., 2010). We also measured the area of the OV in these embryos and found that it was reduced in size to a similar degree in *Jag1* null embryos (*P*<0.05) (Fig. 3). To ensure that differences were not due to changes in the overall embryo size, we also measured the length of the neural tube in these embryos and found that there was no significant difference on average (Fig. 3).

### Dual inactivation of *Tbx1* and *Jag1* causes a synergistic expansion of neuroblasts in the absence of increased cell proliferation

The above data indicate that Tbx1 and Jag1 are expressed in a largely complementary manner in the OV, but there is some overlap in expression in NSD border cells as well as in the posterior OV. Further, loss of *Jag1* results in a 60% reduction in the size of the CVG, opposite to the phenotype resulting from a loss of *Tbx1*. To shed light on the requirement of *Jag1* at the NSD border, as well as to test whether *Tbx1* and *Jag1* might genetically interact, we inactivated *Jag1* within the *Tbx1* expression domain using a knock-in *Tbx1^Cre^* allele.

We first performed *in situ* hybridization using a *Jag1* RNA probe showing that *Jag1* expression was reduced in posterior and, to a lesser extent, anterior domains of the OV at E10.5 in *Tbx1^Cre/+^;Jag1^flox/−^* as well as *Tbx1^Cre/−^;Jag1^floxflox^* mutant embryos, when compared to *Tbx1^Cre/+^;Jag1^flox/+^* and *Tbx1^Cre/−^* littermate control embryos, respectively (Fig. S5). These findings confirmed that *Jag1* was inactivated within the *Tbx1* lineage as expected. Note that *Jag1* expression was also reduced in the pharyngeal apparatus, where the two genes also overlap in expression, but not in regions where they are not co-expressed, such as the forelimb (Fig. S4). It is also important to note that resulting *Tbx1^Cre/+^;Jag1^flox/+^* conditional double heterozygous embryos also have reduced dosage of *Tbx1* since the *Cre* is knocked into the endogenous *Tbx1* locus, producing a functionally null allele (Huynh et al., 2007).

**Fig. 4.**
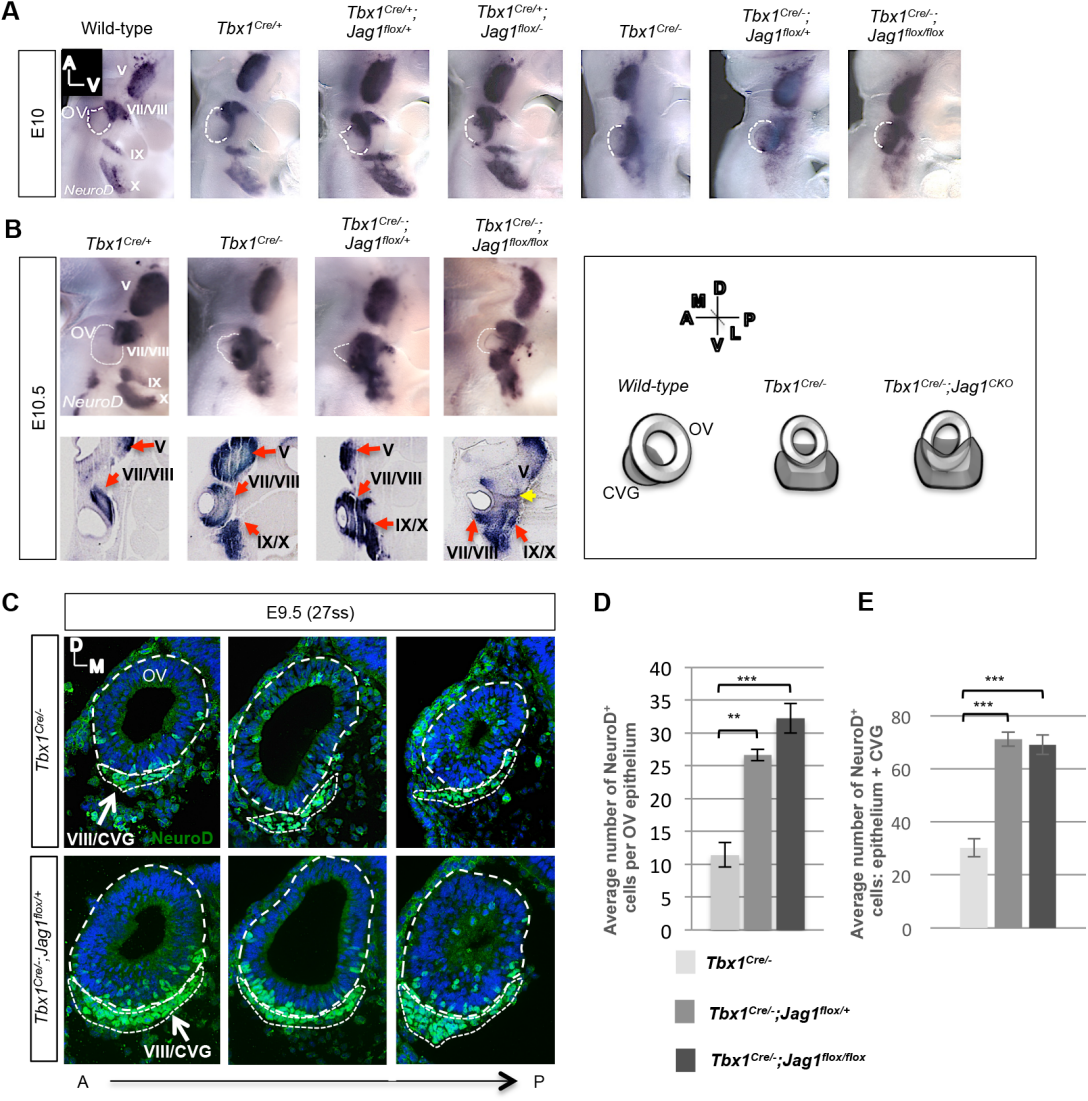
**Inactivation of *Tbx1* and *Jag1* with *Tbx1^Cre^* results in expanded proneural gene expression.** (A,B) Whole-mount *in situ* hybridization was performed on genotypes shown, using an antisense probe for *NeuroD* at E10 (A) and E10.5 (B). There is a synergistic increase in expression in the CVG (cranial ganglion VIII) as *Tbx1* and *Jag1* dosage decreases. There is a similar increase in expression in the other cranial ganglia as well (VII, IX, X), shown in both whole mounts and sections. Fusion of all the ganglia with the Vth ganglion occurs in *Tbx1^Cre/−^;Jag1^flox/flox^* mutants, indicated by the yellow arrow. Adjacent is a schematic depicting the phenotype observed in *Tbx1* single and *Tbx1;Jag1* double mutants. (C) Immunofluorescence on transverse sections with a NeuroD antibody. Both the CVG and OV epithelium are outlined with a white dashed line. The mean total numbers of NeuroD^+^ cells in the OV epithelium alone (D) and the OV epithelium in combination with the CVG (E) were significantly greater in double mutant OVs as compared to *Tbx1^Cre/−^* OVs. Note: three embryos and six OVs per genotype were used for each group. Embryos were stage-matched at 27ss. Data are mean±s.e.m. **P<0.005; ***P<0.001.

To test whether CVG development would be affected in resulting double mutants, RNA *in situ* hybridization was performed for *NeuroD* at E10 and E10.5. At E10, *Tbx1^Cre/+^* embryos exhibited a small, but visible, increase in *NeuroD* expression along the anterior-posterior (A-P) axis of the ventral OV, compared to wild-type controls (Fig. 4A). Double heterozygous *Tbx1^Cre/+^;Jag1^flox/+^* embryos at E10, compared to *Tbx1^Cre/+^* embryos, exhibited a greater expansion in *NeuroD* expression. This expansion was along the dorsal-ventral (D-V) axis of the anterior OV, with the CVG rudiment appearing larger (Fig. 4A). We then inactivated both alleles of *Jag1* by generating *Tbx1^Cre/+^;Jag1^flox/−^* embryos. These embryos exhibited an even greater expansion in *NeuroD* expression along the D-V axis and posterior ventral domain of the OV. A large increase of *NeuroD* expression occurred in *Tbx1^Cre/−^* embryos. In these embryos, an ectopic ganglion formed in the posterior domain of the OV, at the same stages (Fig. 4A,B). Further, the anterior and posterior ectopic CVG became fused, along with cranial ganglia VII and IX (Fig. 4A,B). This phenotype has previously been described (Raft et al., 2004; Xu et al., 2007) and is shown for the sake of comparison. When one or both copies of *Jag1* were inactivated on a *Tbx1^Cre/−^* background, *NeuroD* expression expanded to an even greater degree than in *Tbx1^Cre/−^* embryos at both E10 and E10.5 (Fig. 4A,B). Histological analysis was performed of whole-mount *in situ* hybridization experiments on both E10 and E10.5 embryos, though only sections at E10.5 are shown (Fig. 4B). The histological sections further illustrated the expansion of the *NeuroD* domain, as well as expansion of distal IXth and Xth ganglia, and fusion of these three ganglia with the Vth ganglion, in the case of the double homozygous mutant embryos (Fig. 4B, yellow arrow). Further, the area of NeuroD expression was quantified in tissue sections for both E10 and E10.5 mutant embryos (Fig. S6A). Indeed, we found that the area of expression was significantly greater in double mutant embryos in than in *Tbx1* single mutant embryos at both stages. This expansion appeared to be dose-dependent, as decreasing dosage of *Tbx1* and *Jag1* correlated with increasing expression levels of *NeuroD*.

Because whole-mount *in situ* hybridization is somewhat limited in the resolution of expression it provides, we also performed immunofluorescence studies on tissue sections using an antibody to NeuroD on double conditional null mutant embryos (*Tbx1^Cre/−^;Jag1^flox/+^* and *Tbx1^Cre/−^;Jag1^flox/flox^*) and *Tbx1^Cre/−^* embryos (Fig. 4C). We used this to quantify the number of NeuroD positive (NeuroD^+^) cells within the OV epithelium alone, as well as the total number of NeuroD^+^ cells within the epithelium, plus cells that have delaminated to form the CVG (Fig. 4D,E) at E9.5. Because the epibranchial ganglia appear to fuse with the CVG in these mutants, it is important that we quantify cells within the OV epithelium alone, in order to isolate the otic epithelial-specific function of Jag1 and Tbx1 from potential function in other cranial placodes or ganglia. More distal ganglia can be easily distinguished as discrete patches of cells in many of the transverse sections; therefore, we are confident that a majority of the cells present in the VIIIth ganglion/CVG region are composed of otic epithelial cells. However, a fraction of those ventral-most cells may likely originate from the epibranchial ganglia, and so, two distinct quantifications are needed. The mean total number of NeuroD^+^ cells from both analyses were significantly greater in double mutant OVs as compared to *Tbx1^Cre/−^* OVs (Fig. 4D). We additionally quantified neuroblast numbers in the CVG in double conditional null mutant embryos (*Tbx1^Cre/−^;Jag1^flox/flox^*) and *Tbx1^Cre/−^* embryos using an antibody to Isl1 on serial sections at E10.5 (Fig. S6B). Similarly, we found that the mean total number of Isl1^+^ cells from both analyses were significantly greater in double mutants as compared to *Tbx1^Cre/−^* mutants (Fig. S6B). Interestingly, we noticed from both NeuroD and Isl1 immunofluorescence experiments that neuroblasts preferentially expanded into the lateral domain of the CVG in double mutants (Fig. 4C; Fig. S6B).

**Fig. 5.**
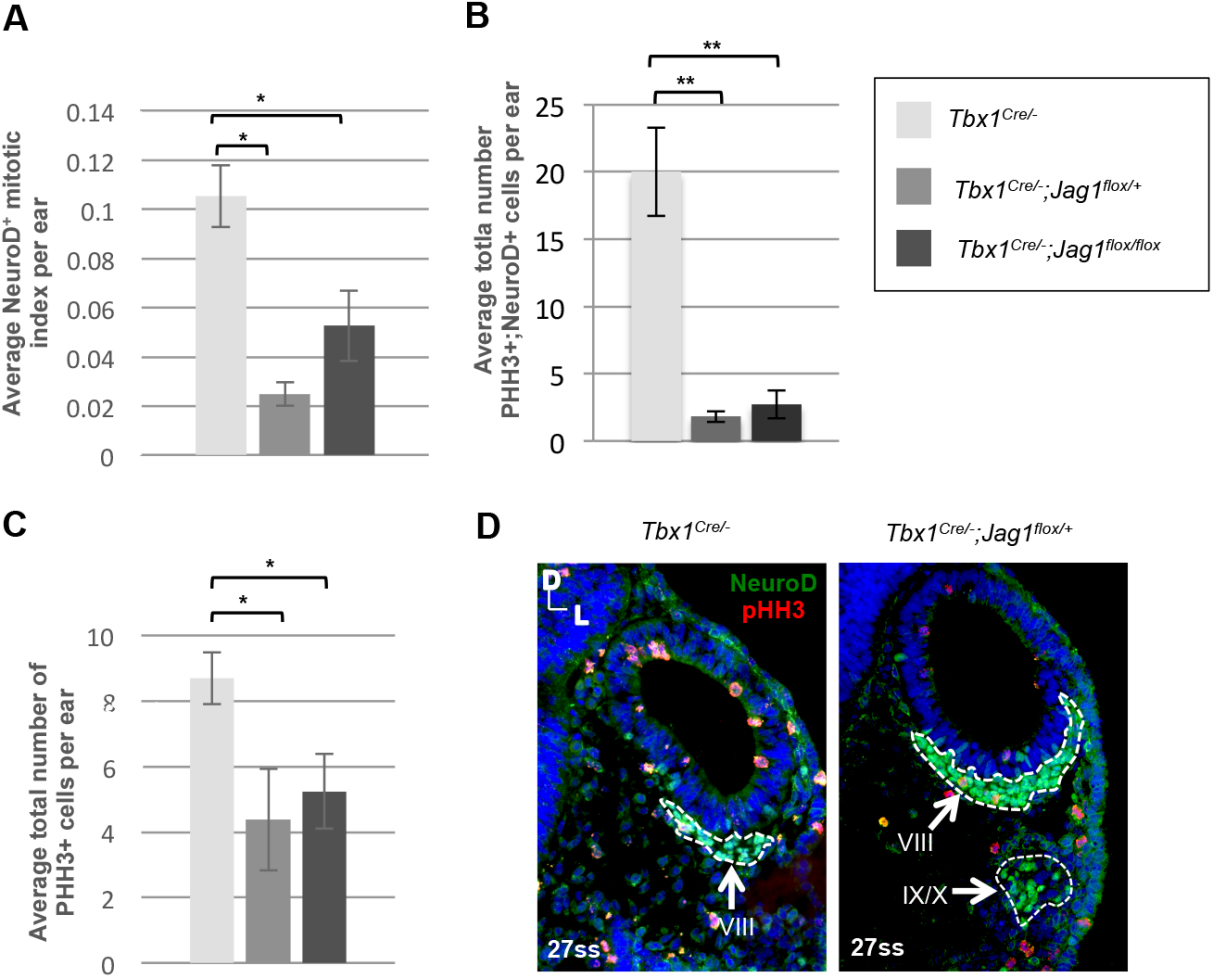
**An increased number of NeuroD^+^ cells is unaccompanied by changes in neuroblast proliferation in *Tbx1^Cre/−^;Jag1^flox/+^* and *Tbx1^Cre/−^;Jag1^flox/flox^* mutants at E9.5.** (A) Bar graphs plotting the mean mitotic index per ear within the neuroblast population as defined by NeuroD expression. Mitotic index was calculated by dividing the total number of proliferating cells (pHH3^+^) that colocalized with NeuroD by the total number of NeuroD^+^ cells. Values were markedly lower in *Tbx1^Cre/−^;Jag1 ^flox/+^* and *Tbx1^Cre/−^;Jag1 ^flox/flox^* OVs and the differences reached statistical significance. (B) Bar graphs plotting the mean total number of pHH3^+^ cells in the OV within NeuroD^+^ domain per OV. Values were statistically significantly lower in *Tbx1^Cre/−^;Jag1 ^flox/+^* and *Tbx1^Cre/−^;Jag1 ^flox/flox^* OVs compared to Tbx1*^Cre/−^* OVs. (C) Bar graphs plotting the mean total number of pHH3^+^ cells in the OV outside the NeuroD^+^ domain per OV. Values were statistically significantly lower in *Tbx1^Cre/−^;Jag1 ^flox/+^* and *Tbx1^Cre/−^;Jag1 ^flox/flox^* OVs compared to Tbx1*^Cre/−^* OVs. (D) Examples of the dual immunofluorescence experiment using antibodies to NeuroD and pHH3, used to calculate the above data. There are visibly fewer proliferating cells throughout the OV of *Tbx1^Cre/−^;Jag1 ^flox/+^* embryos compared to *Tbx1^Cre/^* embryos. The VIIIth ganglion/CVG is markedly larger in *Tbx1^Cre/−^;Jag1 ^flox/+^* mutants compared to *Tbx1^Cre/−^* mutants. A distinct portion of the fused distal epibranchial ganglia (IX/X) is also outlined below. Note: three embryos and six OVs per genotype were used for each group. Embryos were stage-matched at 27ss. Data are mean±s.e.m. *P<0.05; **P<0.005.

One explanation for the expansion of the NSD region in *Tbx1;Jag1* double conditional null mutant embryos, is that there is an increase in cell proliferation of neural progenitor cells. To address this, we performed immunofluorescence on tissue sections at E9.5 using antibodies to phospho-Histone H3 (pHH3) to mark proliferating cells, and NeuroD to mark the neuroblasts (Fig. 5D). We calculated the mitotic index of proliferating neuroblasts from the OV by dividing the total number of proliferating cells (pHH3^+^) that colocalized with NeuroD by the total number of NeuroD^+^ cells, and these numbers were averaged per OV. We found that the mitotic indices were not higher, but in fact they were lower in both *Tbx1^Cre/−^;Jag1^flox/+^* and *Tbx1^Cre/−^;Jag1^floxflox^* embryos compared to *Tbx1^Cre/−^* embryos (Fig. 5A,B). These differences were statistically significant: *P*<0.05 for both. We also compared the average number of pHH3^+^ cells that colocalized with NeuroD per ear and found consistent results (Fig. 5B). Thus, proliferation alone cannot account for the changes in neural precursor expression observed. We also noted that the OV was noticeably smaller in *Tbx1;Jag1* double conditional null mutant embryos compared to *Tbx1^Cre/−^* embryos, and wondered if this could be due to a reduction in the number of non-neural proliferating cells. Indeed, we found that there was a significant reduction (*P*<0.05 for both double mutants) in the average number of proliferating cells that were negative for NeuroD expression within the OV at E9.5 (Fig. 5C). One possibility is that the smaller OV arises due to a change in cell fate from non-neural to neural and, subsequently, cells delaminated from the OV to form the CVG. These findings were somewhat surprising since inactivation of *Jag1* is associated with a decrease in CVG size (Fig. 3) (Pan et al., 2010). Nonetheless, our findings show that the expansion of the neurogenic region observed in *Tbx1*;*Jag1* double mutant embryos is not merely additive, nor synthetic, but rather it is due to a synergistic expansion of neuroblasts when both genes are lost at the NSD border. Taken together, these findings suggest that *Tbx1* and *Jag1* genetically interact at the NSD border to regulate neurosensory patterning across the medial-lateral axis of the OV, and that loss of both genes brings about an overproduction of neuroblasts contributing to the CVG that is not caused by increased cell proliferation.

### Later embryonic defects in the inner ear in *Tbx1* and/or *Jag1* mutant embryos

**Fig. 6.**
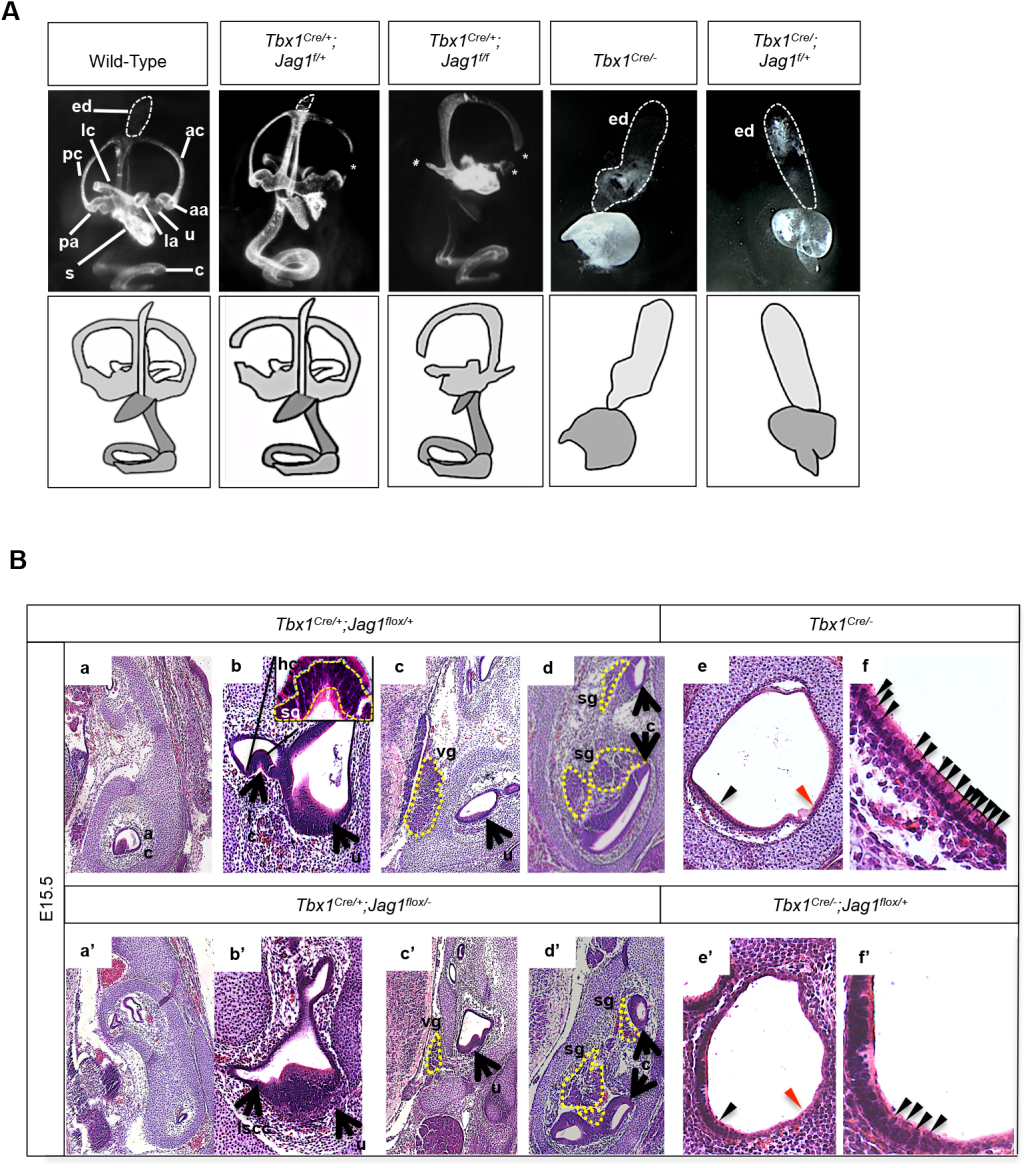
**Gross morphological inner ear defects in *Tbx1*;*Jag1* compound mutant embryos at E15.5.** (A) Paintfilling of E15.5, *Tbx1^Cre/+^;Jag1^flox/+^*, *Tbx1^Cre/+^;Jag1^flox/flox^*, *Tbx1^Cre/−^*, *Tbx1^Cre/^*^−^*;Jag1^flox/+^* and wild-type control inner ears. *Tbx1^Cre/+^;Jag^flox/+^* ears are relatively normal but present with narrowing of the canals, particularly the anterior canal (white asterisk). *Tbx1^Cre/+^;Jag^flox/flox^* ears display incomplete development of the anterior semicircular canal (ac), posterior semicircular canal (pc) and lateral semicircular canal (lc), as well as their associated ampullae, respectively (aa, pa, la) (white asterisks). *Tbx1^Cre/−^* mutant ears have an enlarged endolymphatic duct (ed) (white tracing), while the rest of the inner ear does not fully develop and remains a vesicle with a posterior protrusion. *Tbx1^Cre/−^;Jag^flox/+^* mutant ears are similar to *Tbx1^Cre/−^* ears but the vesicle is smaller and the protrusion is extended ventrally. (B) Transverse histological sections stained by H&E. All three cristae develop normally in *Tbx1^Cre/+^*^;^*Jag1^flox/+^* embryos (a,b). The anterior crista (ac) and lateral crista (lc) attached to the utricle (u) are shown. An image of b at a higher magnification in the inset shows rows of hair cells (hc) and support cells (sc) that develop normally. All three cristae are missing from *Tbx1^Cre/+^;Jag1^flox/flox^* ears (a′,b′). Vestibular (vg) and spiral ganglia (sg) appear to form normally in *Tbx1^Cre/+^;Jag1^flox/+^* embryos (c,d). The vg is noticeably smaller in *Tbx1^Cre/+^*^;^*Jag1^flox/flox^* embryos while the sg appears to develop normally (c′,d′). The inner ear rudiment (the vesicle ventral of the endolymphatic duct) in *Tbx1^Cre/−^* mutants overall are mostly lacking in hair cells, particularly in lateral regions (red arrowhead); however, the medial ventral wall of the vesicle is fairly densely populated with hair cells (black arrowhead) (e). An image of e at a higher magnification is shown in f. *Tbx1^Cre/−^;Jag1^flox/+^* ears are similar to *Tbx1^Cre/−^* ears in that they are mostly lacking hair cells, particularly in lateral regions (red arrowhead) (e′). Medial regions are populated by some hair cells, but much more sparsely. An image of e′ at a higher magnification is shown in f′.

Paintfilling and histological analysis of embryos was performed to examine the morphology of the inner ear during later embryonic stages (Fig. 6; Fig. S7). We first compared *Tbx1^Cre^;Jag1^flox/flox^* and *Tbx1^Cre^;Jag1^flox/−^* embryos at E15.5 to littermate controls. We found that *Tbx1^Cre^;Jag1^flox/flox^* mutant embryos had similar inner malformations as observed when *Jag1* was conditionally inactivated using the *Foxg1^Cre^* allele (Kiernan et al., 2006). The *Foxg1^Cre^* allele is frequently used to inactivate genes throughout the otic epithelium (Hébert and McConnell, 2000), presumably resulting in a broader inactivation of *Jag1* than would a *Tbx1^Cre^*-mediated inactivation. The major phenotype observed was incomplete formation of the SCCs and ampullae, as well as a slightly smaller saccule (Fig. 6). Closer observation with histological analysis on E15.5 and adult inner ears (Fig. 6B, a-d′; Fig. S7), revealed that in *Tbx1^Cre^;Jag1^flox/flox^* or *Tbx1^Cre^;Jag1^flox/−^* mutants, all three cristae were missing and the saccule was shortened. The rest of the sensory structures developed normally. Hair cells in the sensory structures that formed appeared histologically normal (Fig. 6B, a-d′; Fig. S7). Previously published *Foxg1^Cre/+^;Jag1^flox/flox^* mutant embryos (Kiernan et al., 2006) shared the cristae and SCC phenotype; however, they also had abnormal cochleae, utricles and associated hair and support cells. The saccules were slightly smaller as in the *Tbx1^Cre^*-mediated mutant embryos, and hair cells were present. *Tbx1^Cre/+^;Jag1^flox/−^* mutants did not exhibit obvious abnormalities in the cochlea or utricles; however, we did not quantify hair or support cell numbers, which may be required in order to reveal subtle differences. We also examined the spiral/cochlear ganglia and vestibular ganglia, and noticed that while there was no significant difference in size in the spiral ganglia between mutants, the vestibular ganglia were much smaller in *Tbx1^Cre/+^;Jag1^flox/−^* mutants compared to *Tbx1^Cre/+^;Jag1^flox/+^* mutants. Since all three cristae are not present in *Tbx1^Cre^;Jag1^flox/−^* mutants, it is likely that lack of hair cell innervation caused the vestibular ganglion to be either underdeveloped or partially degenerate (Fig. 6B, c,c′).

Next, we examined *Tbx1^Cre/−^;Jag1^flox/+^* embryos compared with *Tbx1^Cre/−^* embryos at E15.5 in the same manner. Paintfilling of *Tbx1^Cre/−^* mutant embryos revealed the endolymphatic duct is greatly enlarged while the rest of the inner ear has been reduced to a vesicle with a small posterior protrusion on the medial side of the ear (Fig. 6A). This is a very similar, though slightly less severe phenotype, to that which has been published (Freyer et al., 2013). Paintfilling of *Tbx1^Cre/−^;Jag1^flox/+^* inner ears revealed a similar phenotype to *Tbx1^Cre/−^* mutants, with an enlarged endolymphatic duct while none of the other structures form (Fig. 6A). The remaining cystic OV in compound mutants was smaller and different in shape compared to *Tbx1^Cre/−^* inner ears. There is also a protrusion from the OV, but it extended ventrally as opposed to posteriorly (*n*=6). Histological analysis of these ears was more telling. Interestingly, although *Tbx1^Cre/−^* ears were severely malformed, the ventral medial portion of the vesicle contained a row of hair cells (Fig. 6B, e,f, black arrowheads). Most of the OV was unpopulated with hair cells outside of this domain (Fig. 6B, e,f, red arrowheads). The *Tbx1^Cre/−^;Jag1^flox/+^* OV contained much fewer hair cells in the same domain (Fig. 6B, e′,f′). Serial sections of mutant embryos confirmed this throughout most of the abnormal OV. In addition, the cochlear and vestibular ganglia were absent in *Tbx1^Cre/−^* and *Tbx1^Cre/−^;Jag1^flox/+^* mutant embryos. Because there are so few hair cells in both mutant embryos, this is again, likely to be due to lack of innervation and, consequently, underdevelopment and/or degeneration of the ganglia and neurons within.

## DISCUSSION

### Tbx1 modulates neurosensory cell fate in a domain-specific manner

**Fig. 7.**
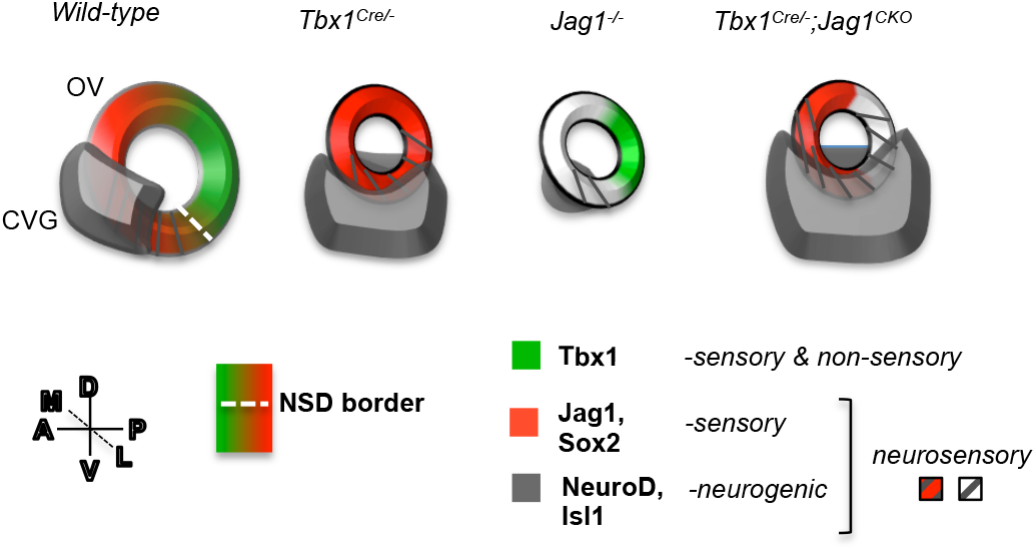
**Model summary.** A schematic of the OV summarizing the expression of the key genes of this study in the OV that is oriented as shown (dorsal-ventral, D-V; medial-lateral, M-L; anterior-posterior, A-P). In wild-type embryos at E10-10.5, Tbx1 is expressed at the NSD border marked by overlapping expression of neurogenic genes (grey stripes) and Jag1/Sox2 (red) in the anteroventral OV. This NSD is positioned adjacent to the developing CVG, marked by NeuroD and Isl1 expression (grey), and contributes to it some of its progenitor cells fated to become neurons. When *Tbx1* is homozygously inactivated (Tbx1^Cre/−^), Jag1 expression is expanded throughout the OV, disrupting the NSD border. The CVG also expands into the posterior and lateral domain in *Tbx1^Cre/−^* (or *Tbx1^−/−^*) OVs. When *Jag1* is inactivated (*Jag1^−/−^*), the Tbx1 expression domain is unchanged, and the CVG is significantly smaller in size. When *Jag1* is inactivated together with *Tbx1* in the *Tbx1* expression domain (*Tbx1^Cre/−^;Jag1^f/+^* or *Tbx1^Cre/−^;Jag1^f/f^* – termed *Tbx1^Cre/−^;Jag1^CKO^*), the NSD border is disrupted and the CVG is further enlarged.

In this study, we found that loss of one allele of *Jag1* in the *Tbx1* expression domain exacerbated the expanded neurogenesis phenotype of *Tbx1* loss-of-function embryos. This co-occurred with increased proneural gene expression in the OV, unaccompanied by changes in cell proliferation. Based upon all this, we created a model that summarizes our findings, shown in Fig. 7. Despite the fact that loss of *Jag1* alone resulted in a smaller CVG than in wild-type embryos, when one or both alleles of *Jag1* was inactivated with *Tbx1*, more neuroblasts were present than in global *Tbx1* null mutant embryos (Fig. 7). This revealed that *Jag1* plays a complex role in neural development that is unmasked when inactivated within the *Tbx1* expression domain together with *Tbx1*, and that *Tbx1* and *Jag1* may genetically interact in this process.

In this work, we found that inactivation of *Tbx1* and *Jag1* within the *Tbx1* expression domain particularly affected the M-L boundary, in addition to the already disrupted A-P boundary seen in *Tbx1*^−/−^ OVs (Fig. 4) (Raft et al., 2004). Relevant to these findings, Sapede et al. (2012) performed cell lineage analysis in zebrafish that identified a population of common progenitors that can give rise to neurons and hair cells that reside in the posteromedial part of the OV. In this study, we found *Jag1* and *Sox2*, required for sensorigenesis and neurogenesis, to be expressed in the posteromedial region of the OV, forming a border with lateral-expressed *Tbx1* at the NSD border. One interpretation of these data is that regulation of the M-L boundary is important for the maintenance of this domain and *Jag1*, along with *Sox2*, serves as marker of this common pool of cells, the fate of which is restricted by *Tbx1*.

*Tbx1* fate mapping in *Tbx1* null mutant embryos also provided evidence suggesting that *Tbx1* may regulate CVG formation in a cell-nonautonomous (as well as cell-autonomous) manner. Based upon these findings, we further speculate that there might be an intermediate gene or genes between *Tbx1* and master proneural transcription factors *NeuroD* and *Ngn1*, one of which may be *Jag1*.

### The role of *Jag1* in NSD development

Our data suggest that *Tbx1* restricts neurosensory cell formation by acting at the NSD border of the OV either directly or indirectly, restricting *Jag1* and *Sox2* expression. Previous findings in chick show that overexpression of human *JAG1* in the OV induces expression of *Sox2* in a Notch-dependent manner (Neves et al., 2011), but that this only occurs for specification of the sensory patches. This study concluded that Sox2 establishes early neurosensory competence*,* which is independent of *Jag1* (Neves et al., 2011). While this is the situation in chick, we speculate that in mouse, *Jag1* plays a role in the specification or maintenance of the fate of cell subtypes within the NSD and not just the sensory patches. Mouse studies showed that *Jag1* is required for the specification of sensory precursors and a subset of neural precursors (Pan et al., 2010). This supports the hypothesis that *Jag1* plays a role in NSD (Pan et al., 2010). Our findings with respect to *Tbx1* also support a new possible role of *Jag1* in the NSD. Furthermore, our studies show that *Jag1* expression may be broader in the mouse OV than in chick and more closely resemble *Sox2* expression (Neves et al., 2011). If the expression of *Jag1* in mouse more closely follows that of *Sox2*, this could indicate that, like *Sox2*, it plays a role in the development of the NSD. Finally, studies in mouse also suggest that the clonal population of progenitors that can give rise to neurons and hair cells may consist of a larger pool of cells than in chick (Fritzsch et al., 2006). If this is the case, regulation of this cell population may necessitate a larger network of genes, such as *Tbx1*. While these are all plausible hypotheses, it is also possible that the differences we see in mouse and chick are due to differences in experimental strategy. Since *Jag1* was overexpressed in chick studies and inactivated in mouse studies, it is possible that *Jag1* is required, but not sufficient, for the development of the NSD.

### *Tbx1* and *Jag1* may be required to maintain a balance of cell types within the NSD

Loss of *Tbx1* may affect neurosensory precursor cell fate via *Jag1*, as evidenced by the further expansion of proneural gene expression when *Jag1* is concomitantly inactivated. Since sensory structures do not develop in *Tbx1*^−/−^ embryos, but the CVG is duplicated (Arnold et al., 2006; Raft et al., 2004; Vitelli et al., 2003), we surmise that the expansion of the area of expression of NSD genes indicates a switch in cell fate from sensory to neural. Additional inactivation of *Jag1* may enhance this switch, based on our findings of a synergistic expansion in proneural gene expression in the OV, as well as failure of cristae and some hair cells to form properly at later stages, in *Tbx1;Jag1* compound mutants. We cannot exclude the possibility that nonsensory cells may also change their fate to form neuroblasts, as the semicircular canals and ampullae also do not fully form in *Tbx1^Cre/+^;Jag1^flox/flox^* mutant inner ears.

Expansion in the neuroblast population observed in the compound mutant embryos appears contradictory to the perceived role of *Jag1* in neurosensory development; however, Notch1 is known to have contrasting roles during neurogenesis and sensorigenesis (Adam et al., 1998; Daudet et al., 2007; Pan et al., 2010; Petrovic et al., 2014), making the interpretation of such experiments complex. One potential explanation for our findings is that *Jag1* may define which cells within the NSD become sensory or neural by repressing neural fate within the boundary cells where it is co-expressed with *Tbx1*. Outside this domain and perhaps earlier in development, *Jag1* may play a role in neurosensory competence together with *Sox2.* This may explain why both the CVG and sensory organs are hypoplastic when *Jag1* is globally inactivated (Pan et al., 2010). Thus, we suggest that *Tbx1* and *Jag1* function in opposing pathways during NSD development, but we speculate that they can also repress neural differentiation within the otic epithelium by acting similarly on common downstream factors.

One of the main strengths of this study is that we had optimal *Cre* alleles to inactivate *Tbx1* and *Jag1* in the same expression domains. This allowed us to discover an interaction between the two genes in neurosensory patterning in which loss of *Jag1* enhances the effect of loss of *Tbx1* in restricting neurogenesis. We performed *in situ* hybridization of many molecular markers but could not observe a difference in the pattern of expression between the *Tbx1* loss-of-function mutant and the double *Tbx1;Jag1* loss-of-function mutant embryos. This is one of the limitations in the study, and we believe this is due to the choice of genes evaluated or lack of resolution of *in situ* hybridization methods we used. To determine the mechanism for the *Tbx1;Jag1* interaction and to gain further insights into cell fate changes in the NSD, we suggest that unbiased genome wide expression profiling approaches are required. This could be coupled with single cell RNA-sequencing of NSD cells in wild-type and mutant OVs in order to fully elucidate the molecular mechanism.

### Conclusions

In conclusion, *Tbx1* and *Jag1* act in discrete regions of the OV to limit the size of the CVG in mouse embryos. Inactivating *Jag1* with *Tbx1^Cre^* reveals a novel role for *Jag1*, whereby in this context *Jag1* acts to repress neural fate, in contrast to its known function. We suggest that normal *Tbx1* and *Jag1* dosage may be required to maintain a proper balance of cell types within the NSD of the OV to form the inner ear. This knowledge could have important implications for future stem cell therapies designed to treat sensorineural hearing loss and vestibular disorders.

## MATERIALS AND METHODS

### Mice

Mice used in this study comply with the regulatory standards of the Institutional Animal Care and Use Committee (IACUC, #20160507) of Albert Einstein College of Medicine. All mouse models have been previously described. The *CMV-GFP* reporter mice were obtained from Dr. Gordon Fishell at New York University Langone Medical Center (Batista-Brito et al., 2009). *Pax2-Cre* mice were provided by Dr. Andrew K. Groves (Ohyama and Groves, 2004). *Tbx1^Cre/+^* mice were provided by Dr. Antonio Baldini (Huynh et al., 2007). *Tbx1*-*GFP* mice, termed ‘*Tbx1 GOF*’, were engineered in our laboratory, in which a Tbx1-GFP fusion protein was added downstream of a loxP-STOP-loxP site in the *Rosa26* locus and used as previously described (Freyer et al., 2013). *Tbx1*^+/−^ and *Tbx1^flox^* mice (Arnold et al., 2006) were engineered in our laboratory and used as previously described. *Jag1* null (stock number 010616), *Jag1^flox^ (*stock number 010618) and *Pax3^Cre/+^* (stock number 005549) mice were obtained from Jackson Laboratories. Embryos were dissected according to date of vaginal plug (E0.5). Embryonic stages <E11.5 were confirmed by counting pairs of somites. Animals were maintained in a 12 h dark/12 h light cycle in compliance with the Albert Einstein College of Medicine Institutional Animal Care and IACUC. Mice were genotyped for *Cre* alleles using the following primers: (5′-CAATGCTGTTTCACTGGTTATG-3′) and (5′-CATTGCCCCTGTTTCACTATC-3′). The *Tbx1^flox^* allele was detected using primers that have been previously described (Braunstein et al., 2009). CMV-GFP reporter and *Tbx1-GFP* mice were genotyped for the GFP allele using the following primers: GFP-Fwd (5′-TAAACGGCCACAAGTTCAGC-3′) and GFP-Rev (5′-GAACTCCAGCAGGACCATG-3′), while the wild-type allele was detected using the following primers: RO1F (5′-GCAATACCTTTCTGGGAGTT-3′) and GFP-wt-R (5′-CAATGCTCTGTCTAGGGGTT-3′). The interval within the *Jag1^flox^* allele was amplified as described by Jackson Laboratories using the following primers: 10092 Jag1 Forward (5′-TCAGGCATGATAAACCCTAGC-3′) and 10093 Jag1 Reverse (5′-CTACATACAGCATCTACATGC-3′). *Jag1* null mice were also genotyped as described by Jackson Laboratories using the following primers: 10089 Forward (5′-TCTCACTCAGGCATGATAAACC-3′), 10090 Wild-type Reverse (5′ TAACGGGGACTCCGGACAGGG-3′), and oIMR8162 Mutant Reverse (5′- TGGATGTGGAATGTGTGCGAG-3′).

### Immunofluorescence on tissue sections

Embryos were fixed in 4% paraformaldehyde at 4°C. Fixation times varied according to embedding method: 2 h for frozen embryos and overnight for paraffin-embedded embryos. Frozen embryos were washed in 0.1 M phosphate buffered saline (PBS) and embedded in 30% sucrose in PBS at 4°C overnight. Embryos were embedded in O.C.T. compound (Tissue-Tek) on dry ice and stored at −80°C. Transverse cryosections were generated at 10 μm in thickness. Tissue sections were washed in PBS and permeabilized in PBS/0.5% Triton X-100 for 5 min. They were then washed in PBS followed by PBS/0.1% Triton X-100 and blocked in 5% goat or donkey serum (G9023 and D9663, Sigma-Aldrich) in PBS/0.1% Triton X-100 for 1 h at room temperature (RT) followed by incubation with primary antibodies diluted in block for 1 h at RT. Primary antibodies used were as follows: rabbit polyclonal α-Tbx1 (1:500; Zymed, San Francisco, CA, USA), goat polyclonal α-Jag1 (1:100; C-20, Santa Cruz Biotechnology), goat polyclonal α-Sox2 (1:500; Santa Cruz Biotechnology), goat polyclonal α-NeuroD (1:500; sc-1084, Santa Cruz Biotechnology), goat polyclonal α-GFP (1:500; Abcam), rabbit polyclonal α-GFP (1:500; ab290, Abcam), rabbit polyclonal α-phospho-histone H3 (1:500; Ser 10, 06-570, Emd Millipore, Billerica, MA, USA), and mouse monoclonal α-Tuj1 (1:1000; MMS-435P, Covance, Princeton, NJ, USA). Tissue sections were then washed three times in PBS/0.1% Triton X-100 and incubated with secondary antibodies diluted in block together with DAPI (4′,6-diamidine-2-phenylidole-dihydrochloride; 1:500) for 1 h at RT. Secondary antibodies were as follows: Alexa Fluor 568 goat α-rabbit IgG (A-11011, Invitrogen), Alexa Fluor 568 goat α-mouse IgG (A-11004, Invitrogen), Alexa Fluor 488 goat α-rabbit IgG (A-11008, Invitrogen), Alexa Fluor 488 donkey α-goat (A-11055, Invitrogen) and Alexa Fluor 568 donkey α-goat IgG (A-11057, Invitrogen). All secondary antibodies were used at a dilution of 1:500. Sections were washed three times in PBS/0.1% TritonX-100 followed by brief washes in PBS and then water. For dual color immunofluorescence, primary antibodies were incubated on tissue sections at the same time, and secondary antibodies were subsequently incubated on sections at the same time. Slides were mounted in Vectashield hard-set mounting medium (H-1400, Vector Laboratories) and stored at 4°C. Images were captured using an Axio Observer (Zeiss, Oberkochen, Germany).

### Whole-mount RNA *in situ* hybridization

Embryos were fixed in 4% paraformaldehyde at 4°C overnight. They were then dehydrated in a series of methanol/PBS/0.1% Tween-20 dilutions to 100% methanol and stored at −20°C. Upon rehydration to 0.1%PBS/0.1% Tween-20, *in situ* hybridization was carried out as previously described (Franco et al., 2001). Antisense digoxigenin-labeled RNA probes to *Tbx1* (Funke et al., 2001), *NeuroD* (Lee et al., 1995), and *Jag1* (Jayasena et al., 2008) were used as described. The *Hes6* RNA probe template was generated from amplified E9.5 mouse cDNA using the following primers: 5′-GGGGAATTAACCCTCACTAAAGGGAACGAGAGT CTTCAGGAGCT-3′ and 5′-GGGGTAATACGACTCACTATAGGGTACAAACGA GGAGCAGCTTC-3′.

### Quantitative analyses

Cell counting and area measurements were performed using ImageJ software. Mitotic index was calculated by dividing the total number of pHH3^+^ cells that colocalized with NeuroD, divided by the total number of NeuroD^+^ cells (within the OV and CVG). OV area was calculated by subtracting the area of the inner OV by the area of the outer OV. Investigators were blinded to group allocation during all analyses. For all analyses, *n*≥6 ears (from at least three different animals) per group. Statistical analysis was performed using Microsoft Excel. Groups were compared using a two-tailed, Independent Samples (unequal variance) *t-*test. Data met the assumption of the test (i.e. data within groups fell under a normal distribution).

### 3D reconstruction

Serial sections of the otic vesicle were aligned using AutoAligner software (Bitplane AG) and regions of interest were traced and reconstructed using BioVis3D software.

### Histology

Embryos were fixed in 4% paraformaldehyde overnight at 4°C. They were then dehydrated to 70% ethanol and embedded in paraffin. Tissue sectioning was performed at 10-12 μm thicknesses. Tissues were cleared in xylene, stained with hematoxylin and eosin (H&E) and then mounted in Permount.

### Paintfilling

Embryos were cut below the forelimbs and fixed in 5% glacial acetic acid, 2% formaldehyde and 75% ethanol overnight. This was followed by dehydration to ethanol and clearing in methyl salicylate. Embryos were bisected dorsally and the brain was removed. A micropipette was used to microinject 0.2% correction fluid diluted in methyl salicylate into the utricle. Paintfilled inner ears were imaged and stored in methyl salicylate.
